# The association between animal-keeping practice and human malaria infection: a cross-sectional study in southern Tanzania

**DOI:** 10.3389/fvets.2026.1794979

**Published:** 2026-07-03

**Authors:** Wei Ding, Longsheng Liu, Yuejin Li, Jinxin Zheng, Shenning Lu, Prosper Chaki, Tegemeo Gavana, Yeromin P. Mlacha, Lulu Huang, Hongmei Li, Yuan Sui, Zhoupeng Ren, Sandra Perez, Roger Frutos, Shan Lv, Xiao-Nong Zhou, Shizhu Li, Peng Bi, Duoquan Wang

**Affiliations:** 1 National Institute of Parasitic Diseases, Chinese Center for Disease Control and Prevention (Chinese Center for Tropical Diseases Research), NHC Key Laboratory of Parasite and Vector Biology, WHO Collaborating Centre for Tropical Diseases, National Center for International Research on Tropical Diseases, Shanghai, China; 2 Université Côte d’Azur, ESPACE UMR 7300, Nice, France; 3 Shandong Institute of Parasitic Diseases, Shandong First Medical University and Shandong Academy of Medical Sciences, Jining, China; 4 School of Global Health, Chinese Center for Tropical Diseases Research, Shanghai Jiao Tong University School of Medicine, Shanghai, China; 5 Ifakara Health Institute, Dar es Salaam, Tanzania; 6 Statistical Center for HIV/AIDS Research and Prevention (SCHARP), Fred Hutchinson Cancer Center, Seattle, WA, United States; 7 State Key Laboratory of Resources and Environmental Information System, Institute of Geographic Sciences and Natural Resources Research, Chinese Academy of Sciences, Beijing, China; 8 Intertryp, UMR 17, CIRAD, Montpellier, France; 9 Faculty of Medicine-Ramatjibodi Hospital, Mahidol University, Bangkok, Thailand; 10 School of Public Health, The University of Adelaide, Adelaide, SA, Australia

**Keywords:** cross-sectional study, malaria, one health, Tanzania, zoopotentiation, zooprophylaxis

## Abstract

**Background:**

Malaria remains a leading global health threat, primarily in sub-Saharan Africa. In Tanzania, malaria continues to cause high morbidity and mortality, especially in rural areas. Animal-keeping, common in these regions, may influence the local malaria vector ecology. However, the relationship between animal-keeping and malaria risk remains to be understood, with studies showing mixed results regarding its protective or risk-enhancing effects. This study assessed the associations between malaria infection and animal-keeping practices in rural southern Tanzania, specifically whether animal species (poultry, ruminants, or pets), management location (indoors vs. outdoors), and herd size influence infection risk, providing insights for targeted malaria control strategies within a One Health framework.

**Methods:**

Data were collected from the baseline survey of the China-Tanzania malaria control project in July 2019, covering three regions: Kibiti, Rufiji and Kilwa. Interviews were conducted with households, including data on living conditions, animal-keeping practices, malaria prevalence, and health-seeking behaviors. Malaria was diagnosed using rapid detection reagents. Univariant analysis and the logistic regression models of exposure-outcome and dosage-outcome were implemented to assess the relationship between malaria infection risk and animal-keeping practices.

**Results:**

Of the 11,406 participants with malaria RDT results, 6,379 (55.93%) kept animals. Poultry-keeping was consistently associated with increased malaria risk across all analyses (univariant analysis OR 1.51 and 1.66, 95% CI 1.35–1.69, 1.51–1.82; exposure-outcome OR 1.409 and 1.596 with 95% CI 1.256–1.580, 1.450–1.757; dosage-outcome OR 1.018 and 1.014, with 95% CI 1.000–1.037 and 1.009–1.018). Results were robust after considering confounding.

**Conclusion:**

Poultry-keeping near households is associated with increased malaria risk in rural southern Tanzania. Findings suggest that managing animal placement and housing could enhance traditional vector control. Adopting a One Health framework that integrates veterinary and environmental management is essential for improving malaria intervention outcomes in rural communities.

## Background

Malaria is one of the major global public health challenges. It is a life-threatening parasitic disease causing by *Plasmodium* and it transmits primarily through *Anopheles* mosquitoes. The World Health Organization (WHO) 2025 World Malaria Report (1) estimated 282 million cases and 610,000 malaria deaths worldwide in 2024. Although global anti-malaria efforts have been continuous, the decrease in global disease burden has stagnated in recent years, particularly in Sub-Saharan Africa (2), which bears the highest burden of malaria, accounting for over 90% of cases and deaths globally.

Tanzania, located in East Africa, is one of the High Burden to High Impact countries listed by the WHO, and it accounts for 3.3% of global malaria cases and 4.3% of global malaria deaths (1). Despite malaria control efforts, the disease remains endemic, particularly in rural regions with inadequate access to healthcare and vector control measures (3). The ability of hematophagous mosquitoes to transmit malaria depends on vector competence, which refers to the biological capacity to acquire, maintain, and transmit *Plasmodium* parasites (31). In Tanzania, malaria transmission primarily occurs by mosquito species within the *Anopheles gambiae* complex and the *Anopheles funestus* group (4, 5). These species exhibit differences in host-feeding preferences. While *An. gambiae sensu stricto* and *An. funestus s.s.* are highly anthropophilic, *An. arabiensis* exhibits more opportunistic feeding behavior and may feed on both humans and animals (6, 7). Such variability in host preference suggests that animal presence around households could influence mosquito feeding patterns and potentially modify human malaria risk (8, 9). In rural Tanzania, animal-keeping is a common practice, integral to the livelihoods of many households. Animals such as chickens, dogs, and ruminants are often kept in close proximity to human dwellings, either indoors or outdoors.

Previous studies have reported mixed findings regarding the role of animal presence in malaria transmission. In some settings, ruminant ownership has been associated with reduced malaria risk (zooprophylaxis), while in others it has been linked to increased transmission (zoopotentiation) (10, 11). These inconsistent findings may reflect differences in vector species, animal-management practices, and spatial proximity between animals and human sleeping areas (12–15). However, many previous studies have examined ruminant ownership at the household level without distinguishing animal species, keeping locations, or exposure intensity. To address these gaps, this study examines multiple categories of animal-keeping practices and differentiates indoor and outdoor exposure as well as animal numbers. This approach allows for a more detailed assessment of how specific animal-keeping practices may influence malaria infection risk (herein measured as *Plasmodium* parasitemia via Rapid Diagnostic Tests (RDT)) in rural Tanzania. This study aimed to assess the associations between malaria infection and different animal-keeping practices among residents in rural southern Tanzania. Specifically, the study examined whether keeping different animal species, namely poultry, ruminants, and pets, and their management location (indoors versus outdoors) were associated with malaria infection risk. In addition, the study explored whether the number of animals kept influenced malaria risk.

## Methodology

### Data background

This study used secondary data collected from the baseline survey database of the China-Tanzania Malaria Control Project conducted between July 2019 and October 2021 in three districts in southeastern Tanzania: Kilwa district in Lindi region and Rufiji and Kibiti districts in Pwani region (5, 16, 17). The selection of the three districts was purposive, considering the logistical convenience and variation in malaria prevalence. Two wards were selected in each district to represent the catchment population, who live within the service area of a particular health center. A multistage sampling approach was used to select households within a village and individuals within a household. The households were enumerated and recruited randomly. In each household, the head of the household was first interviewed, and one other available household member was selected randomly from each of three age groups (<5 years, 5–15 years, >5 years) to participate in the survey. Informed assent and parental consent were obtained for individuals aged 15 or under. For children who could not respond to the survey, the head of the household or the caregiver were interviewed instead (18). A total of 11,406 participants were involved in the project. The data collection for the project included a malaria test and a survey questionnaire on the individual participants’ socioeconomic conditions. Malaria testing was done with RDTs [CareStartTM Malaria Pf/PAN (HRP2/pLDH) Ag Combo, Access Bio, Inc. 65 Clyde Rd., Suite A, Somerset, NJ 08873, United States] at community testing stations. The socioeconomic survey developed based on the Malaria Indicator Survey Toolkit collected data on household characteristics, knowledge, use of malaria preventative measures, health expenditures, and use of health services (19).

### Statistical analysis

#### Variables

Appendix 1 displays all variables used in the study with definitions. In accordance with the study design, the dependent variable was defined as the malaria diagnosis outcome for each participant (positive or negative). The independent variables included a combination of animal-keeping types (any species of animal, namely pets: dog and cat, poultry: chicken and turkey, ruminants: cow, sheep, and goat, or other animals) and animal-keeping locations (indoor, outdoor, or any locations). As evidenced by the existing academic efforts (20–22), the resident’s demographic status, as well as socioeconomic conditions, may have relationships to both animal-keeping behavior and malaria infection, which would be treated as confounders in this study. Therefore, we attempted to cover resident’s demographic and socioeconomic variables as potential confounders, including (1) age, (2) gender, (3) education level, (4) family primary income source, (5) household size, and (6) health insurance ownership. Variables directedly aimed at malaria protection, including (7) use of mosquito nets in the house, (8) window screen protection from mosquitoes, and (9) spray of pesticide in the house, were also included as potential confounders. During the selection of potential confounders, we were aware that this process may act as a source of bias in the multiple regression analysis because the database did not include the full picture describing residents’ demographics and socioeconomic status. For instance, quantitative variables indicating income amount, historical health conditions, religious norms, and ethnic groups were not included.

Essential data cleaning and variable transformation were processed to facilitate the data analysis. First, according to the age cut method adopted by the Tanzanian National Bureau of Statistics (23), we rearranged the age variable into three subgroups of “0–15,” “16–64,” and “>64.” In addition, since more than 90% of the participants held an education level not higher than primary school, education level was regrouped as “illiterate,” “primary school,” and “secondary school or higher.” Furthermore, based on the income source, the family’s primary income source variable was regrouped into “no income or receive donation,” “by other method or casual labor,” “by agricultural (fishing, farming, ruminants keeping),” and “by industries or commercial (skilled labor, driver, salary, business, pension).” The house size variable was also cut into three subgroups according to the variable distribution: “<50m^3^ (first quartile),” “50–143 m^3^ (second-third quartile),” and “>143m^3^ (fourth quartile).” Missing values were found in the family primary income source category (*n* = 41), which were replaced as “other method.”

#### Univariate analysis

To learn the relationship between individual variables and malaria infection, univariate logistic regression analyses were performed multiple times with odds ratio (OR) output and 95% confidence intervals (CI). To learn the difference in malaria prevalence among each subgroup, chi-square tests were performed. For subgroups with less than 40 participants, Fisher’s exact test was performed instead of chi-square tests. Both chi-square tests and Fisher’s exact tests set the alpha at 0.05.

#### Multivariable logistic regression I: practice to malaria infection

A multivariable logistic regression model was built to learn how animal-keeping practices were related to malaria infection. Within the model, the dependent variable was the malaria diagnosis outcome, and the independent variables were animal-keeping practices including (1) keeping pets indoors, (2) keeping pets outdoors, (3) keeping poultry indoors, (4) keeping poultry outdoors, (5) keeping ruminants indoors, (6) keeping ruminants outdoor, (7) keeping other animals indoors, and (8) keeping other animals outdoors. As the practice “keeping the animal any place” referred to the condition where the residents kept their animal indoors or outdoors, to prevent significant collinearity in the multiple logistic models, any “keeping any animal,” and “keep animal anywhere” practice was not included. The candidate confounders were (1) gender, (2) age, (3) education level, (4) primary income source, (5) household size, (6) insurance ownership (7) use of mosquito nets, (8) window protection, and (9) use of pesticide spray. For robust confirmation of the confounders, we adopted the change-in-estimate (CIE) approach (24, 25). The CIE approach is a confounder that captures statistics by comparing the changes in the model’s estimated outcomes before and after the candidate confounders are adjusted. We applied the change-in-estimate (CIE) approach for confounder identification, using a threshold of 10%. If the removal of a candidate confounder from the model caused a relative change of more than 10% in the effect estimate for at least one independent variable, the variable was identified as a confounder. In addition to the CIE approach, Variance Inflation Factor (VIF) tests were also conducted.

We adopted e-value analysis to assess the sensitivity of the multivariable logistic regression model (26). The e-value refers to the unmeasured confounder’s minimal strength of association to both intervention and outcome that can fully explain the intervention-outcome association in a relative risk context. For this study with odds ratio outputs, the e-value was transformed by the formula *Relative Risk ≈ sqrt(Odds Ratio)* (26). By obtaining the e-value, we can learn whether the observed exposure-outcome association can be easily explained by unmeasured confounders. A larger E-value indicates that the unmeasured confounders must have stronger associations to both exposure and outcome to explain the observed associations, in other words, the observed associations are robust, and possible confounders have been adjusted.

#### Multivariable logistic regression II: exposure dosage to malaria infection

We constructed another multivariable logistic regression model to learn the association between the number of animals kept and malaria infection. Starting with the Practice to Malaria Infection model, we replaced the exposure variables from binary (yes or no) to integer (how many), while the candidate confounders remained the same. The CIE method for confounder identification, as well as E-value sensitivity analysis and the VIF analysis, were also conducted for this model. By adopting the Practice to Malaria Infection model and Exposure Dosage to Malaria Infection model in a shared confounder and sensitivity analysis setting, we can make comparative conclusions on the associations between animal-keeping practices and malaria infection by considering the outcomes of the two models together.

## Result

### Descriptive analysis: sociodemographic characteristics of the study population

A total of 11,406 participants were included in the final analysis. The descriptive analysis outcome is shown in Table 1. Regarding animal-keeping practices, 44.07% of the participants (5,027) did not keep any animals. Among those who did (6,379, 55.93%), outdoor animal-keeping was more common than indoor keeping (4,475 vs. 2,321). Poultry was the most frequently kept animal species (6,180 vs. 821, 442, and 97). In terms of demographics, most of the participants were male (8,267, 72.48%), and approximately half were under 16 years old (5,737, 50.29%). In terms of socioeconomic status, 51.26% of the participants were illiterate (5,874), followed by 44.43% of participants with a primary education level (5,068). The primary source of family income was agricultural-related (8,793, 77.13%), including fishing, farming, and ruminant-keeping. Around half of the participants lived in medium-sized houses (5,706, 50.03%). The vast majority of the participants lacked health insurance (10,174, 89.2%). Table 1 also presents the distribution of malaria prevalence across different participant groups. In total, there were 3,101 malaria cases, accounting for 27.19% of the study population.

**Table 1:** Sociodemographic characteristics of the study population and univariate analysis on animal-keeping practices, demographic status, socioeconomic factors, and conventional malaria control practices.

Variables	Subgroups		*n* (%)	Malaria positive (%)	Odds ratio (95 CI)	X^2^ (*p*-value)	X^2^ (*p*-value)
Animal keeping	Does not keep animals		5,027 (44.07%)	1,119 (22.26%)	Reference	Compares to does not keep animals	Within the subgroup
	Does keep animals	Indoor	2,321 (20.35%)	688 (29.64%)	1.47 (1.32, 1.64)	46.67 (<0.05)	4.03 (0.13312)
		Outdoor	4,475 (39.23%)	1,424 (31.82%)	1.63 (1.49, 1.79)	110.43 (<0.05)	
		Any	6,379 (55.93%)	1982 (31.07%)	1.57 (1.45, 1.71)	110.25 (<0.05)	
	Keeps pets: dog and cat	Indoor	278 (2.44%)	63 (22.66%)	1.02 (0.77, 1.37)	0.02 (0.87538)	14.3 (<0.05)
		Outdoor	605 (5.3%)	211 (34.88%)	1.87 (1.56, 2.24)	47.65 (<0.05)	
		Any	821 (7.2%)	256 (31.18%)	1.58 (1.35, 1.86)	31.24 (<0.05)	
	Keeps poultry: chicken and Turkey	Indoor	2,118 (18.57%)	639 (30.17%)	1.51 (1.35, 1.69)	50.26 (<0.05)	5.73 (0.05689)
		Outdoor	4,207 (36.88%)	1,353 (32.16%)	1.66 (1.51, 1.82)	114.52 (<0.05)	
		Any	6,180 (54.18%)	1938 (31.36%)	1.6 (1.46, 1.74)	115.71 (<0.05)	
	Keeps ruminants: cow sheep and goat	Indoor	18 (0.16%)	5 (27.78%)	1.34 (0.48, 3.78)	Fisher (0.57240)	0.73 (0.77782)
		Outdoor	434 (3.81%)	113 (26.04%)	1.23 (0.98, 1.54)	3.26 (0.07089)	
		Any	442 (3.88%)	116 (26.24%)	1.24 (1, 1.55)	3.69 (0.05475)	
	Keeps other animals	Indoor	13 (0.11%)	3 (23.08%)	1.05 (0.29, 3.81)	Fisher (1.00000)	0.04 (1.00000)
		Outdoor	97 (0.85%)	25 (25.77%)	1.21 (0.77, 1.92)	0.68 (0.41049)	
		Any	110 (0.96%)	28 (25.45%)	1.19 (0.77, 1.84)	0.63 (0.42607)	
Gender	Male		8,267 (72.48%)	2,282 (27.6%)	Reference		2.63 (0.10489)
	Female		3,139 (27.52%)	819 (26.09%)	0.93 (0.84, 1.02)		
Age (Year)	0–15		5,737 (50.3%)	2092 (36.47%)	Reference		509.33 (<0.05)
	16–64		4,966 (43.54%)	914 (18.41%)	0.39 (0.36, 0.43)		
	>64		703 (6.16%)	95 (13.51%)	0.27 (0.22, 0.34)		
Education level	Illiterate		5,847 (51.26%)	1,638 (28.01%)	Reference		37.32 (<0.05)
	Primary school		5,068 (44.43%)	1,388 (27.39%)	0.97 (0.89, 1.05)		
	Secondary school or higher		491 (4.3%)	75 (15.27%)	0.46 (0.36, 0.6)		
Family income source	No income or receives donation		388 (3.4%)	80 (20.62%)	Reference		61.81 (<0.05)
	By other method or casual labor		107 (0.94%)	20 (18.69%)	0.89 (0.51, 1.53)		
	By agriculture (fishing, farming, ruminant keeping)		8,797 (77.13%)	2,548 (28.96%)	1.57 (1.22, 2.02)		
	By industries or commercial (skilled labor, driver, salary, business, pension)		2,114 (18.53%)	453 (21.43%)	1.05 (0.8, 1.37)		
House size (M^3^)	<50 (first quarter)		2,853 (25.01%)	874 (30.63%)	Reference		34.52 (<0.05)
	50–143 (second to third quarter)		5,706 (50.03%)	1,552 (27.2%)	0.85 (0.77, 0.93)		
	>143 (fourth quarter)		2,847 (24.96%)	675 (23.71%)	0.7 (0.63, 0.79)		
Insurance	Has insurance		1,232 (10.8%)	210 (17.05%)	Reference		71.77 (<0.05)
	No insurance		10,174 (89.2%)	2,891 (28.42%)	1.93 (1.66, 2.25)		
Mosquito net	Household applied mosquito net		10,464 (91.7%)	2,814 (26.9%)	Reference		5.40 (0.0201)
	No mosquito net		942 (8.3%)	287 (30.5%)	0.84 (0.73, 0.97)		
Window protection	Window protected by mosquito screen		3,721 (32.6%)	791 (21.3%)	Reference		97.65 (<0.05)
	Window not protected by mosquito screen		7,685 (67.4%)	2,310 (30.1%)	0.63 (0.57, 0.69)		
Pesticide spray	House sprayed pesticides		489 (4.3%)	104 (21.3%)	Reference		8.73 (<0.05)
	House does not spray pesticides		10,917 (95.7%)	2,997 (27.5%)	0.71 (0.57, 0.89)		
Total			11,406 (100.00%)	3,101 (27.19%)			

### Univariate analysis

The last three columns of Table 1 present the outcomes of univariate analysis. For animal-keeping practices, keeping animals indoors, outdoors, or in any location was significantly associated with an increased risk of malaria infection (OR, respectively, 1.47, 95% CI 1.32–1.64, 1.63, 95% CI 1.49–1.79, 1.57, 95%CI 1.45–1.71). Compared with non-animal keepers, malaria prevalence was significantly higher among those who kept animals indoors, outdoors, or in any location (chi-square test, *p* < 0.05 for each group). However, no significant difference in prevalence was observed among the indoors, outdoors, and any-location keeping groups when compared with each other (chi-square test, *p* = 0.13). For pet (dogs and cats) owners, keeping pets outdoors (OR = 1.87, 95% CI: 1.62–2.24) or in any location (OR = 1.58, 95% CI: 1.35–1.86) posed a significantly higher risk of malaria compared to non-animal keepers risk factor (chi-square test *p*-value <0.05). In contrast, keeping pets indoors showed no evidence of being a risk factor (OR 1.02, 95% CI 0.77–1.37), and there was no difference in malaria prevalence compared to no animal-keeping residents (chi-square test *p*-value 0.88). Among the subgroups who kept pets, a statistical difference was found in malaria prevalence (chi-square test p-value <0.05). Keeping poultry indoors, outdoors, or both was identified as a risk factor with ORs of 1.51 (95% CI 1.35–1.69), 1.66 (95%CI 1.51–1.82), and 1.60 (95% CI 1.46–1.74), respectively. The three practices presented significant malaria prevalence differences compared to no animal-keeping (chi-square test *p*-value <0.05), but there were no differences between the three practices (chi-square test *p*-value 0.06). Keeping ruminants in any place was suggested to be a risk factor (OR 1.24, 95%CI 1–1.55) while keeping ruminants indoors and outdoors lacked significance (OR1.34, 1.23, 95% CI 0.48–3.78, 0.98–1.54). No significant malaria prevalence was found in the ruminants-keeping category compared to those who did not keep animals (Fisher’s exact test *p*-value 0.57, chi-square test p-value 0.07 and 0.54); the difference between the category was also not significant (chi-square test p-value 0.78). Possibly due to sample insufficiency, none of the univariant analyses for keeping other animal practice was significant (OR 1.05, 1.21, 1.19, 95% CI 0.29–3.81, 0.77–1.92, 0.77–1.84; Fisher’s exact test *p*-value 1, chi-square test p-value 0.41, 0.42, 1).

In terms of demographic status, statistics suggested being a woman was not significantly associated with a reduced risk of malaria (OR 0.93, 95% CI 0.84–1.02), and the malaria prevalence among the two genders presented limited differences (chi-square test p-value 0.10). However, older age was identified as a protective factor: for those aged 16–64 and older than 64, risks for malaria infection were 61% (OR 0.39, 95% CI 0.36–0.43) and 73% (OR 0.27, 95% CI 0.22–0.34) lowered compared to those younger than 16. There were also significant differences in malaria prevalence among the three age groups (chi-square test *p*-value <0.05).

According to the univariant analysis for socioeconomic factors, having an education level of secondary school or higher showed a protective effect (OR 0.46, 95% CI 0.36–0.60), while primary school level presented limited significance (OR 0.97, 95% CI 0.89–1.05). Malaria prevalence differed significantly across education-level subgroups (chi-square test *p*-value <0.05). For the family’s primary income source, statistical outcomes suggested that agriculture was a greater risk factor for malaria infection (OR 1.57, 95% CI 1.22–2.02) than no income or receiving donations, while insufficient evidence supported the effects of other methods of income or casual labor (OR 0.89, 95% CI 0.51–1.53) and industrial or commercial jobs (OR 1.05, 95% CI 0.80–1.37). A difference in malaria prevalence was found among the family primary income source subgroups (chi-square test *p*-value <0.05). Compared to those living in small houses (<50 m^3^), larger houses were identified as a protective factor for malaria infection, as middle-sized house (50–143 m^3^) showed a 0.85 OR (95% CI, 0.77–0.93), and a large house (>143 m^3^) presented a 0.70 OR (0.63–0.79). The prevalence of malaria also differed among three house-size groups (chi-square test p-value< 0.05). The absence of health insurance was identified as a risk factor for malaria infection (OR 1.93, 95% CI 1.66–2.25). Among the health insurance subgroups, malaria prevalence differed significantly (chi-square test *p*-value <0.05).

For the conventional malaria control practices of mosquito net use, window protection, and pesticide use, the results of univariant analysis indicated a common protection effect with odds ratios of 0.84 (95% CI 0.73–0.97), 0.63 (95% CI 0.57–0.69), and 0.71(95% CI 0.57–0.89), respectively. Significant differences in within-group malaria prevalence were found in window protection and pesticide spray groups (chi-square p-value both <0.05).

### Multivariable logistic regression I: practice to malaria infection

The CIE approach to identifying confounders was conducted prior to the multiple regression model building. The output of the CIE approach is provided in Appendix 2. Table 2 presents the final outcomes of the CIE approach. The candidate confounder, family primary income source, was identified to be associated with one exposure variable, with an average percentage change of 49.2%. Appendix 3 presents the VIF test for multicollinearity of the variables in the model, indicating a low multicollinearity of the build.

**Table 2:** Confounders identified by the CIE approach of the logistic regression model for associations between practice and malaria infection.

Confounder	Number of exposures affected	Exposures affected	Average % change
Income	1	Ruminants indoors	49.2
Age	5	Pets indoors, ruminants indoors, ruminants outdoors, other animal indoors, other animal outdoors	37.9
Education level	3	Ruminants indoors, ruminants outdoors, other animal outdoors	31.8
Mosquito net	3	Ruminants indoors, ruminants outdoors, other animal outdoors	26.4
House Size	4	Ruminants indoors, ruminants outdoors, other animal indoors, other animal outdoors	52.7
Insurance	4	Ruminants indoors, ruminants outdoors, other animal indoors, other animal outdoors	52.4
Gender	2	Ruminants indoors, ruminants outdoors	29.1
Pesticide spray	3	Ruminants indoors, ruminants outdoors, other animal outdoors	107.6
Window protection	5	Pets outdoors, poultry indoors, ruminants indoors, other animal indoors, other animal outdoors	46

The candidate confounders of education level, use of mosquito nets, gender, and pesticide spray affected, respectively, 3, 3, 2, and 3 exposure variables, with average changes of 31.8, 26.4, 29.1, and 107.6%. Both age and window protection affected five independent variables, with average percentage changes of 37.9 and 46.0%. According to the 10% confounder identification criteria, we treat these candidate variables as confirmed confounders, adjusting for them in the following multivariable logistic regression model.

**Figure 1:**
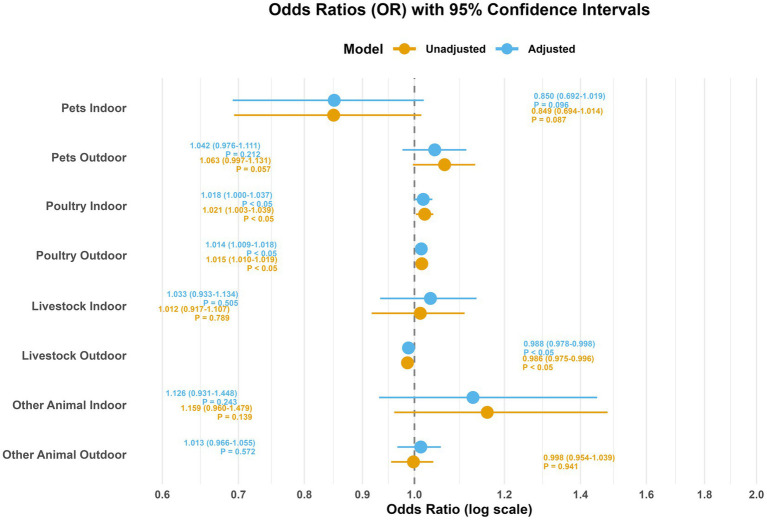
Result of the logistic regression model for association between practice and malaria infection.

The output of the logistic regression model is presented in Appendix 4 and Figure 1. The unadjusted logistic regression model did not include the confirmed confounders, while the adjusted logistic regression model did. The interpretation of the multivariable logistic regression will be based on the adjusted model. Keeping pets indoors was identified as a protective factor for malaria infection (OR 0.73, 95% CI 0.544–0.981), while keeping pets outdoors was a risk factor (OR 1.23, 95% CI 1.02–1.48). The indoor and outdoor poultry-keeping practices were both risk factors, with ORs, respectively, of 1.409 and 1.596 (95% CI 1.25–1.58 and 1.45–1.76). According to the model output, there was insufficient statistical evidence to suggest an effect from indoor and outdoor ruminants keeping on malaria infection due to a 95% CI of 0.37–3.13, and 0.65–1.04. There was also no significance from keeping other animals indoors and outdoors for malaria infection (95% CI 0.18–2.57 and 0.56–1.45).

Appendix 5 shows the E-value analysis outcomes. The model shows robustness for the following three reasons. Firstly, the E-value analysis was contextualized with a comprehensive inclusion of detected confounders (age, gender, education level, family primary income source, house size, insurance ownership, bed nets use, window screens, and pesticide spray). Secondly, all E-values were larger than the effects point-estimated of the matched variables For instance, for the variables ‘keep pets indoors,’ ‘keep poultry indoors,’ ‘keep poultry outdoors,’ and ‘keep other animals indoors,’ the E-values exceeded both their corresponding ORs and 2. This indicates that any unmeasured confounder would need to have odds ratios greater than the observed ORs and greater than 2. in its associations with both the exposure variable and malaria infection to fully explain the associations estimated by the model. Thirdly, in the condition where the ORs approached the lower 95% boundary, the e-values were still larger, presenting an extreme context in which the unobserved confounders must have stronger effects than the estimate OR when the observed OR is close to zero.

### Multivariable logistic regression II: exposure dosage to malaria infection

**Figure 2:**
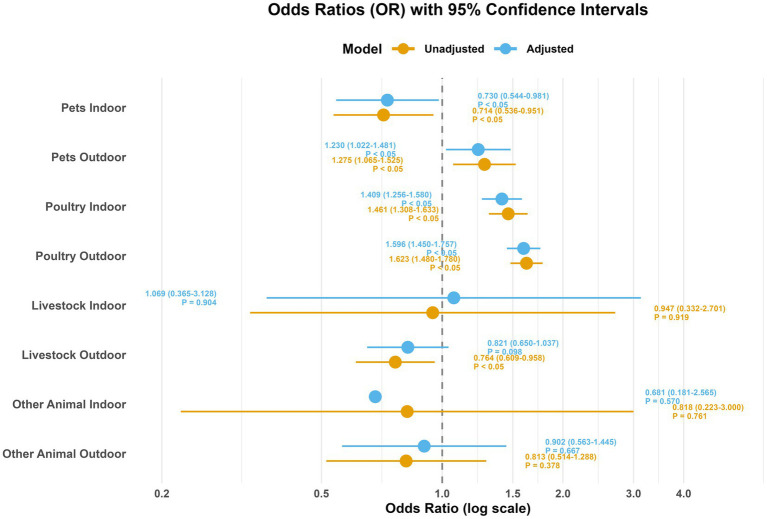
Result of the logistic regression model for association between exposure dosage and malaria infection.

Table 3 shows the CIE analysis of the multivariable logistic regression model for the association between exposure dosage and malaria infection. The confounder identification ensured consistency with the practice-infection model with all candidate confounders confirmed to be confounders (income, age, education level, use of bed nets, house size, insurance, sex, pesticide spraying, and window protection). Details of the CIE for the model are attached in Appendix 6 and the VIF test output for low multicollinearity in Appendix 7. The output of the model is presented in Appendix 8 and Figure 2. Statistical significance was found for keeping poultry indoors, poultry outdoors, and ruminants outdoors. The output indicated that each poultry kept indoors would lead to a 1.8% increase in malaria infection risk (OR 1.018, 95% CI 1.000–1.037), each additional poultry kept outdoors would cause a 1.4% malaria infection risk increase (OR 1.014, 95% CI 1.009–1.018). Each ruminant kept outdoors would lead to a 1.2% risk deduction (OR 0.988, 95% CI 0.978–0.998). The outcomes of the E-value analysis for the model are shown in Appendix 9. Even though the E-values were smaller compared to the exposure-outcome model, it still indicated the model’s robustness since the e-values were larger than the ORs and the variables were integers referring to the numbers of changes.

**Table 3:** Confounders identified by the CIE approach of the multivariable logistic regression model for the association between exposure dosage and malaria infection.

Confounder	Number of exposures affected	Exposures affected	Average % change
Income	1	Ruminants outdoors	13.01
Age	2	Ruminants outdoors, other animal outdoors	58.69
Education	2	Ruminants outdoors, other animal outdoors	11.98
Mosquito net	2	Ruminants outdoors, other animal outdoors	13.68
House size	4	Pets indoors, ruminants indoors, ruminants outdoors, other animal outdoors	13.41
Insurance	4	Pets indoors, ruminants indoors, ruminants outdoors, other animal outdoors	22.89
Gender	2	Ruminants outdoors, other animal outdoors	15.75
Pesticide spray	3	Ruminants indoors, ruminants outdoors, other animal outdoors	20.07
Window protection	5	Pets indoors, pets outdoors, poultry indoors, other animal indoors, other animal outdoors	34.05

## Discussion

By triangulating results from the univariant analysis and two multivariable logistic regression models (exposure-outcome and dosage-outcome), we found that the three analyses showed significant association between poultry-keeping practices and malaria infection. Across all three analytical approaches, both indoor and outdoor poultry-keeping were associated with increased malaria risk (univariant analysis OR 1.51 and 1.66, 95% CI 1.35–1.69, 1.51–1.82; exposure-outcome OR 1.409 and 1.596 with 95% CI 1.256–1.580, 1.450–1.757; dosage-outcome OR 1.018 and 1.014, with 95% CI 1.000–1.037 and 1.009–1.018). However, for indoor and outdoor keeping practices, including pets, ruminants, and other animals, the three analyses did not present consistency in indicating the risk and significance. Therefore, we were convinced that indoor and outdoor poultry-keeping practice was associated with malaria infection, acting as a risk factor for the disease.

This result supports previous evidence from the Democratic Republic of the Congo, where household chicken ownership was associated with higher *P. falciparum* prevalence in settings dominated by anthropophilic *An. gambiae* (15). One proposed explanation is that olfactory and volatile compounds secreted by chickens might attract *An. gambiae s.s*., the common vector in the DR Congo; thereby increasing mosquito density in peri-domestic environments. In addition, the living conditions for chickens might provide additional mosquito breeding habitats, particularly when hygiene and waste management are suboptimal.

In contrast, animal-keeping practices involving pets, ruminants, and other animals did not demonstrate consistent associations with malaria infection across analytical models. Nevertheless, the dosage-outcome analysis revealed a statistically significant protective effect of outdoor ruminants keeping, with each additional ruminant kept outdoors associated with a 1.2% reduction in malaria risk (OR 0.988, 95% CI 0.978–0.998). This finding aligns with studies conducted in Zambia, the Democratic Republic of Congo, Ethiopia, the Gambia, and southern Tanzania, which provide evidence for ecological interactions between ruminants and malaria vectors (8, 12–15, 27, 28). In southern Tanzania, for example, *Anopheles arabiensis* has been observed to rest preferentially in cattle sheds when ruminants are present and to enter human dwellings in their absence. Moreover, households with ruminants have been shown to exhibit reduced human blood indices for *An. arabiensis* and *An. funestus s.l.*, suggesting a diversion of mosquito bites from humans to animals (27, 28).

These findings highlight the importance of considering animal management practices within malaria control strategies. From a One Health perspective, animal husbandry practices may influence vector behavior and human exposure by modifying host availability and peri-domestic ecological conditions. For example, zooprophylaxis can be an effective malaria control strategy when mosquitoes preferentially feed on ruminants over humans, as seen with *An. arabiensis*, an opportunistic feeder with a tendency toward ruminants where available (6), or improving poultry housing structures to reduce vector attraction to households. Integrating animal management guidance with existing malaria interventions such as insecticide-treated nets and environmental vector control may provide additional opportunities for reducing malaria transmission in rural communities where close human–animal interactions are common (9, 29, 30).

Several limitations of this study should be acknowledged. First, this study did not collect entomological data such as vector species composition, mosquito density, or host-feeding preferences. As a result, the proposed ecological mechanisms linking animal-keeping practices and malaria risk cannot be directly confirmed in this study and should be interpreted cautiously. Future studies incorporating entomological surveys and vector blood-meal analyses would be valuable for clarifying the biological pathways underlying these associations. Second, malaria diagnosis was based on RDTSs, which may produce false-negative or false-positive results. Third, the study sites were purposively selected for logistical feasibility, which may introduce selection bias and limit the generalizability of the findings to other ecological or socioeconomic contexts. Fourth, small sample sizes for certain animal categories, such as cows, ducks, and turkeys, limited statistical power and may have contributed to non-significant findings. Fifth, household GPS coordinates were not analyzed in this study, therefore potential spatial autocorrelation between households was not accounted for in the regression models, which should be considered a direction for future research. Finally, the cross-sectional design restricts causal inference, and temporal relationships between animal-keeping practices and malaria infection cannot be conclusively established.

## Conclusion

This study provides evidence that keeping poultry near households is associated with increased malaria risk in rural southern Tanzania. These findings suggest that animal management practices may influence malaria transmission through ecological interactions between humans, animals, and mosquito vectors. Incorporating guidance on animal husbandry, such as poultry housing management and ruminant placement, into malaria prevention programs may complement existing vector control strategies. More broadly, the results support the value of a One Health approach that integrates human health, veterinary practices, and environmental management to improve control of vector-borne diseases in rural communities.

## Data Availability

The datasets presented in this article are not readily available because access to this dataset is subject to the signing of a data sharing agreement between the requestor’s institution and our research team. Requests to access the datasets should be directed to wangdq@nipd.chinacdc.cn.

## In memoriam

The late Dr. Prosper Chaki made foundational contributions to the China-Tanzania Malaria Control Project. We honor his memory and dedication, which made this dataset and study possible.
